# Time and Temperature: Changes in Heat-Related Mortality over 27 Years

**DOI:** 10.1289/ehp.123-A287

**Published:** 2015-11-01

**Authors:** Wendee Nicole

**Affiliations:** Wendee Nicole was awarded the inaugural Mongabay Prize for Environmental Reporting in 2013. She writes for *Discover*, *Scientific American*, *National Wildlife*, and other magazines.

Predictions of how climate change will affect public health^1^ depend on accurate projections of future risk. Several recent studies suggest the risk of mortality associated with extreme heat has declined with time.^2^ But methodological and data differences between these studies have made it difficult to fully elucidate what’s now known to be a complex relationship between temperature and health. A study reported in this issue of *EHP* expands this line of research by applying consistent methodology across a broader geographic scope, including countries with different climates and demographic profiles.^3^

The authors analyzed data from multiple cities and nations using two-stage time-series modeling, including a meta-analysis. The data set included more than 20 million deaths from nonaccidental causes in seven countries: Australia, Canada, Japan, South Korea, Spain, the United Kingdom, and the United States. The team used mean daily temperature as the exposure index, with data back to 1985 in some locations. They restricted the analysis to the four warmest months of the year for each location.

**Figure:**
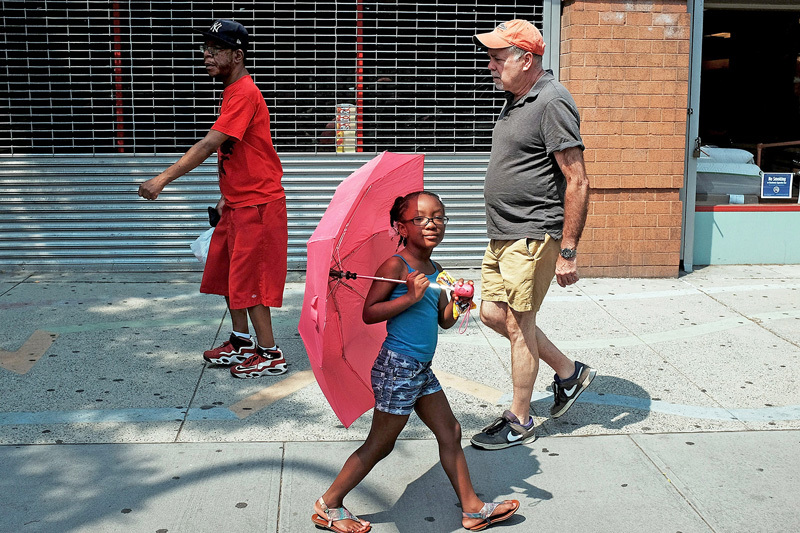
The estimated risk of heat-related mortality fell over time in more than half the countries studied, possibly a result of factors such as better housing and healthcare, increased use of air conditioning, and public education. © Spencer Platt/Getty Images

Results showed that the estimated relative risk of heat-related mortality significantly declined with time in the United States, Japan, and Spain, but the authors found no evidence of a decline in relative risk in the United Kingdom. Canada had a nonsignificant decrease in heat-related mortality, and low statistical power for South Korea and Australia made the results difficult to analyze for those countries.^3^

“Little is known about which individual or population-level factors modify the association between heat and mortality,” says lead author Antonio Gasparrini, senior lecturer in biostatistics and epidemiology at the London School of Hygiene & Tropical Medicine. Reasons for the lowered mortality were not analyzed in this work. However, the authors suggest the reduction could be due to improvements in infrastructure, such as better housing and healthcare, increased use of air conditioning, and education about the risks of extreme heat.^3^

Air conditioning prevalence has increased over time in four of the countries where Gasparrini and colleagues reported a decrease in heat-related mortality (Canada, Japan, Spain, and the United States), but has stayed the same in one of the countries where there was no change in mortality over time (the United Kingdom).^3^ Studies conducted elsewhere have found evidence of reduced mortality associated with higher use of air conditioning across time or geographical regions,^4^^,^^5^ although others have found only a weak association.^6^

“The study design is appropriate and technical analysis quite elegant,” says Joacim Rocklöv, an associate professor of epidemiology at Umeå University in Sweden, who was not involved with the study. “However, the aim of the study is temperature and not climatic extreme heat waves, which can be harder to study in shorter time periods but which can show different trends in effects not captured here.” Furthermore, Rocklöv says, pooling at the country level can mask considerable within-country differences.^7^

It is unclear why the authors found no decline in heat-related mortality in the United Kingdom after the severe European heat wave of 2003, whereas they did in Spain. Gasparrini suggests this could be due to lack of more current data from the United Kingdom. He explains that Spain’s heat adaptation plans after the 2003 heat wave may have provided benefits that were measurable in the Spanish data, which extended to 2010. In contrast, U.K. data covered only the years 1993–2006, with only three post-2003 years.^3^

Rocklöv also points out that the study includes only high-income countries where factors such as urbanization and housing standards differ from locations where billions of other people live.

One limitation of several heat-related mortality studies has been that the heat–mortality relationship is assumed to change in a linear fashion over time. “The authors emphasize how their approach flexibly models the association between temperature and mortality, allowing for a nonlinear relationship as well as delayed effects,” says Jennifer Bobb, assistant scientific investigator at Group Health Research Institute, who was not involved with the current study. “And this is indeed a strength of the analysis.” Bobb says relaxing this linear assumption also enables the investigation of a broader range of questions, such as whether most of the decline occurred early or late in the study period, or whether the decline in risk has slowed over time.
